# The Digital Renaissance of Customized Subperiosteal Implants—Clinical Outcomes, Surgical Workflow, and Current Evidence for the Rehabilitation of Severely Atrophic Jaws: A Narrative Review

**DOI:** 10.3390/dj14100685

**Published:** 2026-10-20

**Authors:** Ioan Sîrbu, Vladimir Năstasie, Andreea Mihaela Custură, Elisei Adelin Radu, Ruxandra Stănescu, Alexandra Tuță, Dana Catrinel Binzaru-Ispas, Gabriela Tănase, Valentin Daniel Sîrbu

**Affiliations:** 1 Discipline of Implanto-Prosthesis Therapy, Faculty of Dentistry, “Carol Davila” University of Medicine and Pharmacy, 37 Dionisie Lupu Street, District 2, 020021 Bucharest, Romania; 2 Doctoral School, Faculty of Dentistry, “Carol Davila” University of Medicine and Pharmacy, 050474 Bucharest, Romania; 3 Discipline of Dental Prostheses Technology, Faculty of Dentistry, “Carol Davila” University of Medicine and Pharmacy, 37 Dionisie Lupu Street, District 2, 020021 Bucharest, Romania

**Keywords:** subperiosteal implant, CAD/CAM, additive manufacturing, 3D printing, atrophic jaw, digital workflow, dental rehabilitation, custom-made implant

## Abstract

**Background/Objectives**: Rehabilitation of the severely atrophic edentulous jaw remains one of the most demanding challenges in oral and maxillofacial reconstruction, particularly when extensive bone augmentation is contraindicated, has previously failed, or is refused by the patient. Subperiosteal implants, first introduced in the 1940s and largely abandoned due to poor long-term performance, have re-emerged over the past decade as a viable alternative through the integration of cone-beam computed tomography (CBCT)-based virtual planning, computer-aided design/computer-aided manufacturing (CAD/CAM), and additive manufacturing. This review summarizes current evidence on the clinical outcomes, surgical workflow, and indications of customized, digitally designed subperiosteal implants. **Materials and Methods**: A structured literature search was performed in PubMed and Scopus, supplemented by manual searching, to identify publications relevant to the predefined clinical and conceptual scope of this narrative review. Search terms addressed subperiosteal implants, digital/CAD-CAM workflows, additive manufacturing, severe jaw atrophy, and clinical outcomes. Predefined selection criteria were applied to maintain consistency with the scope of the review, and the literature-selection process was documented using a descriptive flow diagram. **Results**: Sixty-two publications were retained as the core literature informing the narrative synthesis, comprising systematic reviews and meta-analyses, retrospective and multicenter clinical studies, case series, case reports, and biomechanical/technical investigations. The synthesized evidence indicates short-to-medium-term implant survival generally exceeding 90%, with soft-tissue exposure and dehiscence as the most frequently reported complications, and a decline in survival on longer follow-up. Digitally designed subperiosteal implants are increasingly reported for severely resorbed maxillae and mandibles, oncologic and post-traumatic defects, syndromic patients, and cases of failed conventional or zygomatic implant therapy. **Conclusions**: Digital workflows have renewed clinical interest in subperiosteal implants by enabling patient-specific design and digitally supported planning and manufacturing. However, the evidence base remains dominated by case reports and heterogeneous retrospective series, underscoring the need for prospective, standardized, long-term studies.

## 1. Introduction

Severe atrophy of the edentulous maxilla or mandible remains a major reconstructive challenge in modern implant dentistry and oral and maxillofacial surgery. Extensive resorption of the alveolar and, in advanced cases, basal bone frequently precludes the placement of conventional root-form endosseous implants without prior bone augmentation. Grafting procedures—including onlay grafting, sinus elevation, guided bone regeneration, and distraction osteogenesis—are associated with prolonged treatment times, unpredictable graft resorption, donor-site morbidity, and increased complication rates in medically compromised or previously irradiated patients. Zygomatic implants have offered a graftless alternative for the atrophic maxilla, but their use is technically demanding, anatomically constrained, and not free of long-term biological and prosthetic complications [1].

Subperiosteal implants were initially introduced in the 1940s as a method of supplying prosthetic support directly from the residual bone surface without necessitating an adequate bone volume for conventional endosseous anchorage. In the past, they were made using direct impressions of the remnant bone that had been surgically exposed, followed by laboratory casting of metallic frameworks. Their steady decline was multifactorial, even though these implants were a significant therapeutic alternative for patients with severely resorbed jaws [2,3,4]. Inaccuracies during bone impression, laboratory casting, and subsequent framework location could jeopardise passive adaptation between the implant and the underlying bone, and the traditional workflow was intrusive and technically difficult [4]. Unfavourable biomechanical conditions, framework instability, increasing bone loss, and eventually implant failure may result from such disparities as well as the restricted capacity to optimise framework shape and load distribution [2,3,4]. Mucosal dehiscence, gradual exposure of the cobalt–chromium (Vitallium) framework, and persistent inflammation and infection were among the equally significant biological consequences [2,3,4]. Crucially, long-term clinical data showed that as follow-up increased, typical subperiosteal implants’ performance declined. Yanase et al. found that implant loss persisted over time, with estimated survival rates of 79% after 10 years and 60% at 15 years in a prospective survival analysis [5]. Concerns about the reliability of traditional subperiosteal rehabilitation in comparison to osseointegrated endosseous implant therapy were also reaffirmed by long-term clinical findings [6]. Therefore, rather than a single dominant reason, the historical decline of subperiosteal implants should be regarded as the product of interacting industrial, biomechanical, and biological limits [2,3,4,5,6].

**Figure 1:**
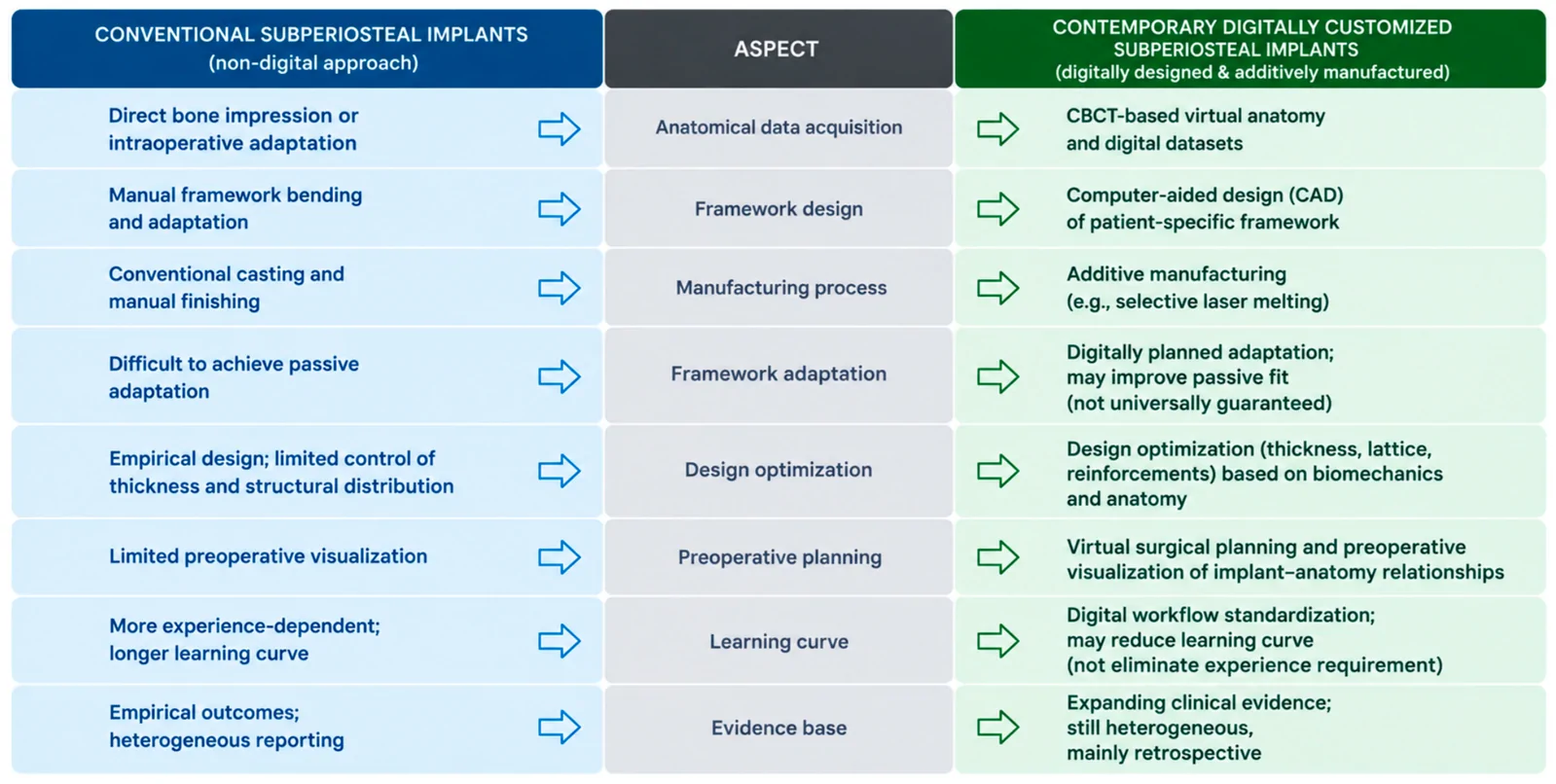
Evolution from conventional non-digital to contemporary digitally designed and additively manufactured subperiosteal implant workflows. The schematic illustrates the transition from direct bone impression, manual framework design, and conventional casting toward CBCT-based anatomical acquisition, CAD-based patient-specific design, virtual planning, and additive manufacturing. The comparison represents general technological trends and should not be interpreted as evidence of superior clinical outcomes of contemporary workflows (the illustration was generated with the assistance of ChatGPT (OpenAI) and subsequently reviewed and adapted by the authors for scientific accuracy).

However, the method has had a “digital renaissance” in the last ten years. Direct bone imprints are no longer necessary thanks to CBCT-based three-dimensional imaging, and patient-specific framework design based on the rebuilt bone anatomy is made possible by virtual surgical planning and CAD/CAM technology [3,4]. In comparison to earlier impression- and casting-based workflows, additive manufacturing technologies, specifically direct metal laser sintering (DMLS) and selective laser melting (SLM) of titanium alloys, further enable the fabrication of customised frameworks with improved precision and adaptability [3,4,7] (Figure 1). These developments have revitalised clinical interest in subperiosteal implants and made their production a patient-specific, digitally controlled process. However, since soft-tissue dehiscence and framework exposure continue to be among the most commonly reported complications of modern customised subperiosteal implants, technological advancements should not be seen as having resolved all biological issues related to the procedure [2,3].

This shift from analog to digital fabrication has renewed clinical and scientific interest in subperiosteal implants as a graftless, immediately loadable solution for the severely atrophic jaw [8,9]. Contemporary customized subperiosteal implants (CSIs) are designed on virtually planned, patient-specific frameworks that are manufactured in one piece or in modular segments and are increasingly proposed not only for the classic indication of severe edentulous atrophy, but also for the reconstruction of oncologic, post-traumatic, and congenital or syndromic defects, and for patients in whom conventional or zygomatic implant therapy has previously failed [10,11,12].

Despite this growing body of literature, the evidence supporting digitally designed subperiosteal implants remains fragmented, consisting predominantly of case reports, small case series, and a limited number of systematic reviews and meta-analyses, several of which have highlighted marked heterogeneity in implant design, manufacturing protocol, follow-up duration, and outcome reporting [13,14]. A comprehensive, up-to-date synthesis of the available clinical and technical evidence is therefore warranted to inform clinical decision-making and to identify gaps that should guide future research.

The modern revival of customised subperiosteal implants has been thoroughly documented in several recent reviews. While Vaira et al. documented the growing range of patient-specific subperiosteal implants across established and developing indications, Albash et al. gave a cutting-edge review of their technological development, materials, digital workflows, and clinical applications [15,16]. The quick growth of this literature, however, also poses a clinically significant question that goes beyond outlining the technologies and indications that are currently available: to what degree have modern digital workflows truly overcome the constraints that contributed to the historical decline of subperiosteal implants, and which issues still prevent their consistent use in severely atrophic jaws? Therefore, a targeted evaluation of the connection between clinical outcomes, long-term consequences, technical advancements, and the quality of the supporting data is necessary.

Therefore, the purpose of this narrative review was to critically assess modern customised subperiosteal implants intended for the rehabilitation of severely atrophic jaws, with a focus on how much digital planning and manufacturing have addressed the shortcomings of earlier subperiosteal implant therapy. The review summarises clinical outcomes, surgical workflows, materials, and complications. It also critically looks at which technological limitations seem to have been overcome, which biological and clinical challenges still exist, how these complications are currently handled, and which evidence gaps still prevent standardised patient selection and wider clinical adoption.

## 2. Materials and Methods

This study was designed as a narrative review supported by a structured literature search. The search strategy and predefined selection criteria were used to enhance transparency and consistency in identifying publications relevant to the predefined clinical and conceptual scope of the review and were not intended to constitute an exhaustive systematic evidence-retrieval process. Accordingly, the identified literature was qualitatively synthesized and critically interpreted within a narrative framework rather than evaluated according to systematic- or scoping-review methodology [17].

A structured literature search was conducted in PubMed/MEDLINE and Scopus. The final database search was performed in June 2026, and the contemporary literature search covered publications from 2021 to 2026. Two electronic databases, PubMed and Scopus, were searched, and the results were supplemented by a manual search of the reference lists of relevant articles and recent issues of specialized journals. The search strategy combined terms related to the intervention (“subperiosteal implant” OR “subperiosteal implants”) with terms related to the digital workflow (digital OR “3D printing” OR CAD/CAM OR custom-made OR “patient-specific”), the target population (“atrophic maxilla” OR “atrophic mandible” OR “severe jaw atrophy”), and the outcomes of interest (complications OR survival OR outcomes), combined with Boolean operators. These databases were selected to provide complementary coverage of the biomedical and dental literature (PubMed) and the broader multidisciplinary and citation-indexed literature (Scopus), consistent with the narrative scope of the review. To increase search sensitivity and identify potentially relevant studies not retrieved electronically, database searching was supplemented by manual screening of the reference lists of relevant publications and recent issues of specialized journals.

To maintain consistency with the predefined scope of the narrative review, publications considered for the core narrative synthesis were selected according to the following criteria: (1) explicit focus on subperiosteal implants; (2) relevance to a digital workflow (CAD/CAM design, CBCT-based planning, or additive manufacturing); (3) relevance to severe jaw atrophy or an analogous reconstructive indication; (4) reporting of clinical outcomes, survival, and/or complications; (5) publication in a peer-reviewed, English-language journal. Records that were non-English or were not focused on subperiosteal implants were excluded. Records retrieved from PubMed and Scopus were exported to Microsoft Excel (Microsoft Corporation, Redmond, WA, USA). Duplicate records were identified primarily by matching DOI and article title. Records without a DOI were additionally compared using title, first author, publication year, and journal, and potential duplicates were manually verified before removal.

**Figure 2:**
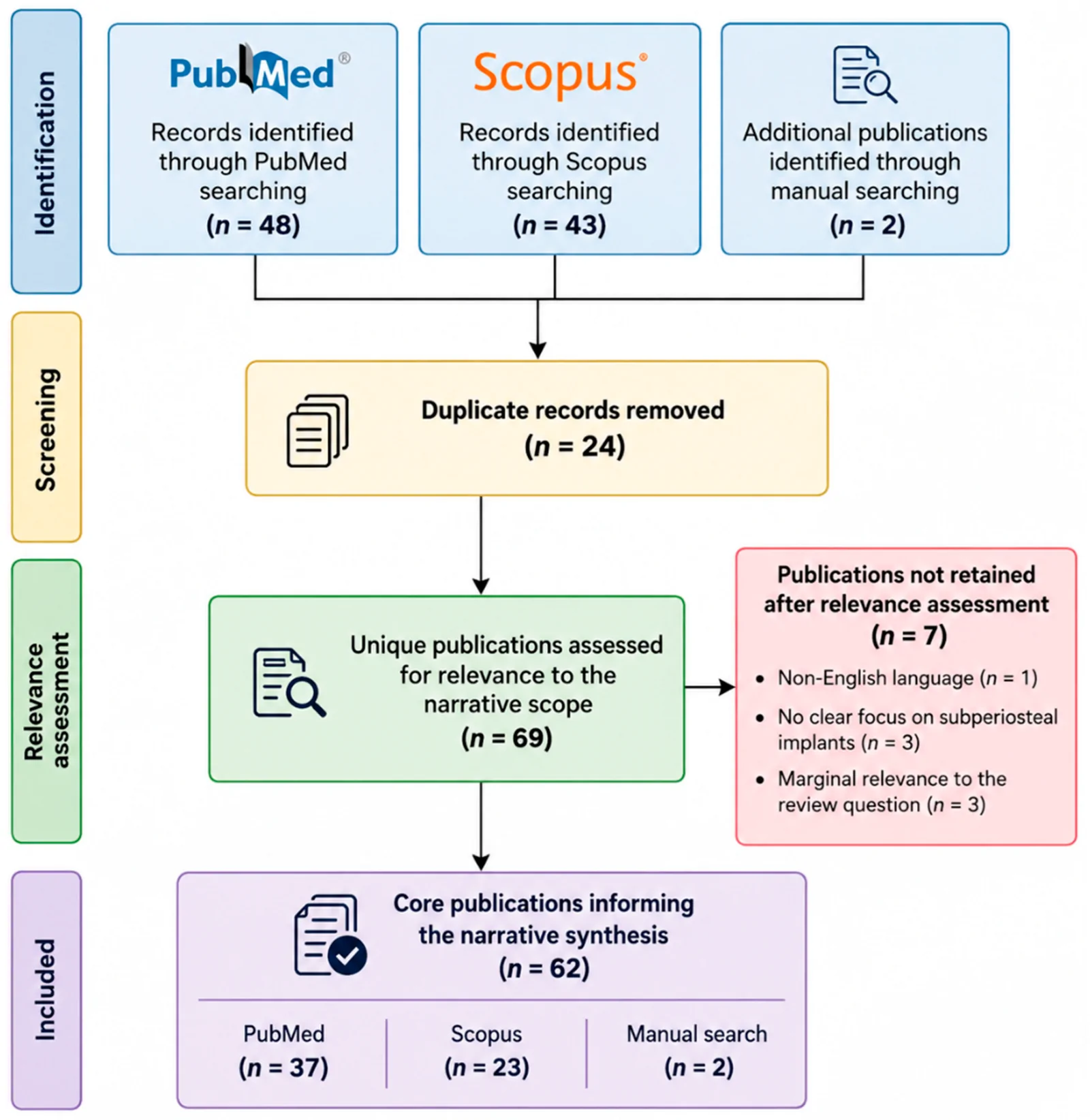
Descriptive flow diagram of the literature-identification and selection process used to define the core publications informing the narrative synthesis.

Records identified through PubMed and Scopus were assessed for relevance to the predefined narrative scope, and duplicate records were removed before further assessment. The literature-identification and selection process is summarized descriptively in Figure 2. The structured search initially identified 93 records (PubMed, *n* = 48; Scopus, *n* = 43; manual search, *n* = 2). After removal of 24 duplicate records, 69 unique publications remained and were assessed for relevance to the scope of the review. Seven publications were not retained because of non-English language (*n* = 1), lack of a clear focus on subperiosteal implants (*n* = 3), or marginal relevance to the review question (*n* = 3). Consequently, 62 publications constituted the core literature informing the narrative synthesis (PubMed, *n* = 37; Scopus, *n* = 23; manual search, *n* = 2) (Figure 2). In addition to the 62-publication core corpus, a limited number of supplementary references were cited outside the predefined literature-selection process for specific contextual, comparative, or recently published evidence. These included the historical publications by Yanase et al. [5] and Schou et al. [6], used to provide historical context regarding the long-term performance and limitations of conventional subperiosteal implants; two recent reviews used to contextualize the present review in relation to contemporary developments in customized subperiosteal implantology; and two recently published sources addressing soft-tissue dehiscence and its clinical management. These supplementary publications were not included in the 62-publication core corpus or in Supplementary Table S1, and were not counted in the descriptive synthesis of the predefined literature set.

Title and abstract relevance assessment and subsequent full-text relevance assessment were performed by one author. Given the narrative design of the review, these procedures were not conducted independently in duplicate. Publications for which relevance to the predefined scope was uncertain were discussed with a second author, and the final decision was reached by consensus. For the purpose of thematic organization, relevant information from the selected publications was charted according to study design, publication year, target region (maxilla and/or mandible), digital workflow characteristics (CBCT-based planning and CAD/CAM design), manufacturing technology and implant material, reported clinical indication, implant survival, and complications, where available. The charted information was subsequently reviewed for consistency and completeness.

To account for differences in evidence type and methodological role, the selected literature was organized into three categories: (1) primary clinical evidence, including prospective and retrospective clinical studies, cohort and multicenter studies, case series, case reports, and other clinical reports; (2) secondary and contextual evidence, including systematic reviews, meta-analyses, scoping reviews, narrative reviews, consensus reports, and perspective articles; and (3) technical and preclinical evidence, including technical reports, finite-element analyses, biomechanical investigations, materials studies, and other non-clinical investigations. These categories were considered separately in the narrative synthesis and were not treated as equivalent sources of clinical evidence. Technical and preclinical publications were used to inform the interpretation of digital workflow, manufacturing, materials, and biomechanical considerations and were not considered clinical outcome evidence.

Potential overlap between primary and secondary evidence was explicitly considered during synthesis. When systematic reviews or meta-analyses included primary studies that were also represented individually in the selected literature, their findings were treated as secondary evidence and were not combined with the corresponding primary-study data as independent observations. Pooled survival or complication estimates reported by secondary studies are therefore presented only as findings of the respective review and are explicitly attributed to their source. Where multiple publications could be identified as originating from the same or potentially overlapping clinical cohort, they were considered non-independent when interpreting clinical outcomes.

Given the heterogeneity of publication types, follow-up periods, and outcome reporting across the selected literature, the evidence was organized into predefined thematic domains and synthesized narratively rather than quantitatively. No formal risk-of-bias assessment or meta-analysis was undertaken as part of the present review. Where systematic reviews or meta-analyses discussed within the narrative synthesis reported pooled outcome estimates, these are presented as such and explicitly attributed to the corresponding source.

To enhance transparency and allow verification of the literature-selection process, the complete search strategy, together with the publications assessed during the selection process and the predefined criteria applied to determine their inclusion in the narrative synthesis, is provided in Supplementary Table S1. The supplementary table documents the study-level selection process and provides an explicit record of the manually conducted eligibility assessment.

## 3. Results

### 3.1. Overview of the Literature Informing the Narrative Synthesis

Among the 62 publications constituting the core literature informing the narrative synthesis, 38 were classified as primary clinical evidence, 19 as secondary or contextual evidence, and 5 as technical/preclinical evidence. The primary clinical literature was dominated by case reports and small case series describing patients treated with digitally designed subperiosteal implants [18,19,20,21,22,23,24,25,26,27,28,29,30,31,32,33,34,35,36,37,38,39,40,41], together with retrospective, multicenter, cohort, pilot, and other clinical studies reporting larger patient series or clinical outcome data [7,42,43,44,45,46,47,48,49,50,51,52,53]. Secondary and contextual evidence comprised systematic reviews, meta-analyses, and scoping reviews [1,2,8,10,12,13,14,54,55], as well as narrative or literature reviews, consensus reports, perspective articles, and mixed review–clinical publications [3,4,11,56,57,58,59]. Publications combining a literature review with case reports or case series were classified within the secondary/contextual domain to avoid counting the same publication in more than one evidence category. Technical and preclinical evidence comprised finite-element, biomechanical, materials, and methodology-focused investigations without independent clinical outcome datasets [60,61,62,63,64]. The distribution of the core literature according to methodological role is summarized in Table 1.

### 3.2. Digital Workflow: CBCT-Based Planning and CAD/CAM Design

**Figure 3:**
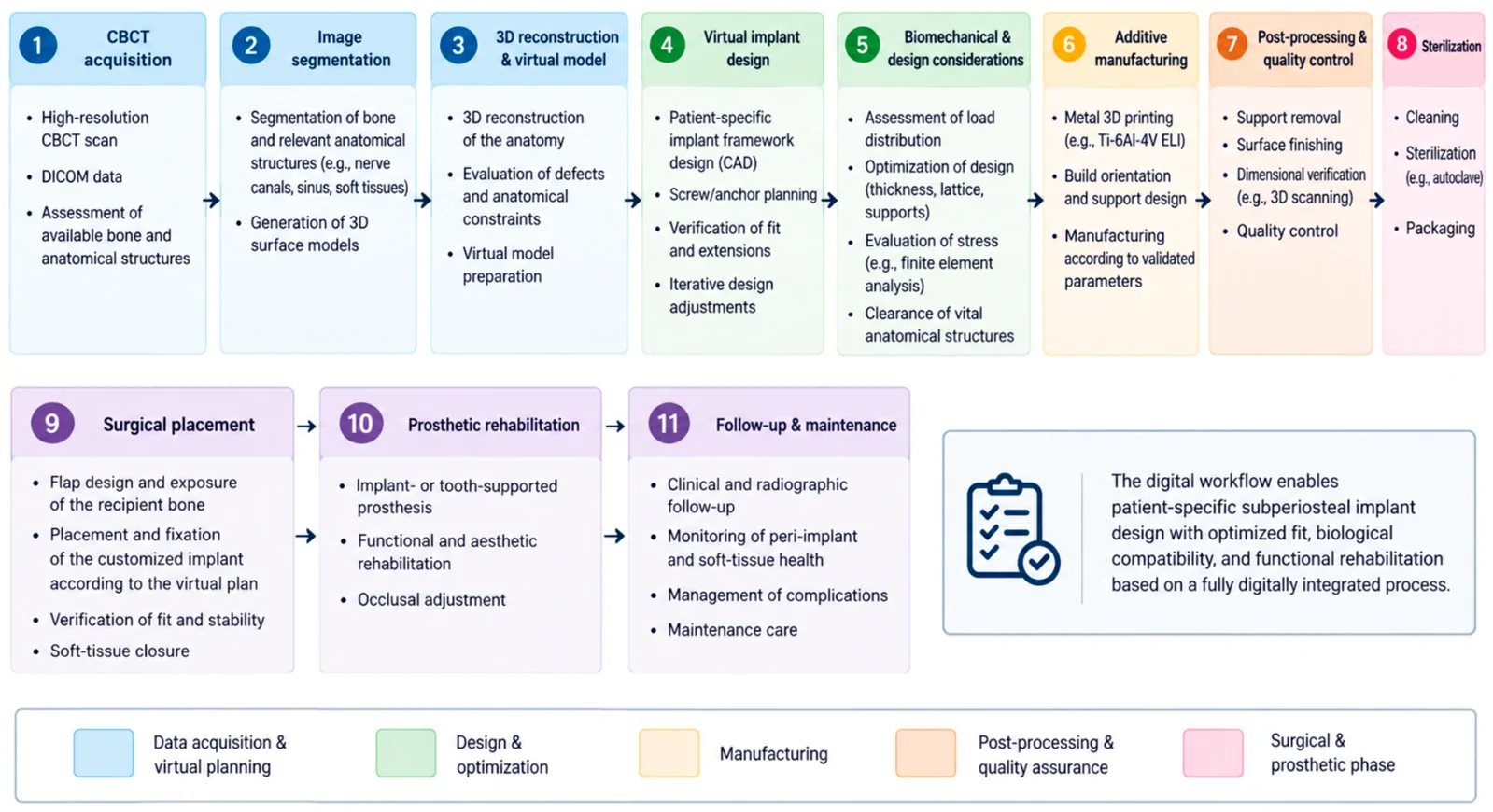
Contemporary digital workflow for patient-specific subperiosteal implant rehabilitation. The schematic summarizes the principal stages from CBCT acquisition and image segmentation to virtual design, manufacturing, surgical placement, prosthetic rehabilitation, and follow-up (the illustration was generated with the assistance of ChatGPT (OpenAI) and subsequently reviewed and adapted by the authors for scientific accuracy).

Across nearly all included studies, the digital workflow followed a broadly consistent sequence: acquisition of a CBCT scan of the atrophic jaw, segmentation of the bone surface, virtual design of a patient-specific framework directly on the reconstructed bone model, and, in most protocols, virtual planning of the prosthetic restoration prior to implant manufacture [3,9,10,65]. This “backward-planning” approach, in which the prosthetic outcome informs the surgical and framework design, was repeatedly emphasized as central to achieving a passive framework fit and predictable prosthetic loading [12,25,51]. Across nearly all included studies, the digital workflow followed a broadly consistent sequence comprising CBCT acquisition, image segmentation and three-dimensional reconstruction, virtual design of a patient-specific framework, manufacturing and post-processing, surgical placement, prosthetic rehabilitation, and subsequent clinical follow-up (Figure 3) [3,52].

Intraoperative verification of the virtually planned design, either through surgical guides or direct visual/tactile confirmation of framework adaptation, was consistently reported as an essential step before definitive fixation [30,37,53]. Some protocols additionally incorporated immediate or early prosthetic loading directly on the digitally designed framework, shortening the overall treatment timeline compared with historical two-stage subperiosteal implant protocols [38,51].

### 3.3. Manufacturing Technologies and Materials

Titanium alloy manufactured through DMLS or SLM was the material most frequently reported across the included studies, reflecting its established biocompatibility, mechanical strength, and long track record in maxillofacial reconstruction [37,39,40,43,47,64]. Alternative polymer platforms, polyetheretherketone (PEEK) and polyetherketoneketone (PEKK), were reported in a smaller number of studies, primarily for their radiolucency, reduced weight, and elastic modulus closer to that of bone [41,60]. One experimental study explored yttria-stabilized zirconia as an additively manufactured alternative, reporting on the design optimization required to achieve adequate mechanical performance in a ceramic material [62], while another investigated lattice-type framework architectures intended to improve osseous integration of the framework with the residual ridge [63].

DMLS and SLM are powder-bed fusion additive manufacturing technologies in which a three-dimensional CAD model is converted into successive layers and fabricated through laser-induced consolidation of titanium or titanium-alloy powder [4,39,47,64]. Although the term “sintered titanium” is frequently used in the clinical literature, it should be interpreted cautiously, as SLM involves complete melting and subsequent solidification of the metallic powder rather than conventional solid-state sintering [66,67]. The layer-by-layer manufacturing process allows the fabrication of complex, patient-specific geometries that would be difficult to reproduce using conventional manufacturing methods, including thin anatomical frameworks, fixation arms, and customized prosthetic connections [4,39,47,63]. However, the final characteristics of additively manufactured titanium may be influenced by powder characteristics, layer thickness, build orientation, laser parameters, and post-processing procedures, which can affect dimensional accuracy, surface roughness, residual stresses, porosity, and mechanical behavior [66,67].

Compared with additive manufacturing, subtractive manufacturing produces the framework by computer numerical control (CNC) machining from a prefabricated titanium block or disc [66,67]. This approach offers a homogeneous starting material, predictable bulk mechanical properties, high dimensional accuracy, and relatively smooth machined surfaces. However, milling is constrained by tool diameter and accessibility, particularly when reproducing undercuts, thin interconnected structures, complex fixation extensions, and highly irregular anatomical geometries, and it is associated with greater material wastage [66,67]. In contrast, additive manufacturing provides greater geometric freedom and more efficient material utilization, making it particularly suitable for customized subperiosteal frameworks adapted to complex residual bone anatomy [4,39,63]. Its limitations include a generally rougher as-built surface and the potential for process-dependent porosity, anisotropy, residual stresses, and dimensional variation, which may necessitate post-processing and precision machining of critical interfaces [66,67]. Therefore, additive and subtractive manufacturing should be regarded as technically distinct rather than hierarchically superior approaches: milling offers advantages in material homogeneity and surface finish, whereas additive manufacturing provides greater design freedom for complex patient-specific geometries [66,67].

Although improved geometric freedom and anatomical adaptation provide a strong rationale for additive manufacturing in customized subperiosteal implants, the available clinical literature does not currently demonstrate that additive manufacturing per se results in superior long-term clinical outcomes compared with subtractive fabrication, as clinical performance is also influenced by framework design and adaptation, fixation, soft-tissue management, prosthetic loading, and maintenance [2,3,13,14].

Finite-element analyses among the included studies examined the biomechanical behavior of subperiosteal frameworks under functional loading, comparing stress distribution across different materials and framework thicknesses, and generally supporting the mechanical suitability of digitally optimized titanium designs while highlighting the need for adequate framework thickness and arm distribution to avoid stress concentration [54,60,61].

### 3.4. Clinical Indications

The predominant indication reported across the included literature was rehabilitation of the severely atrophic, fully or partially edentulous maxilla and/or mandible in patients considered unsuitable for, or unwilling to undergo, extensive bone augmentation [4,45,46]. A substantial subset of studies reported use of customized subperiosteal implants as a salvage or alternative solution following failure of conventional endosseous or zygomatic implant therapy [21,57].

Beyond the classic atrophic-edentulism indication, digitally designed subperiosteal implants were increasingly reported for the reconstruction of complex oncologic and post-ablative defects, including total or subtotal maxillectomy, frequently in combination with free-flap reconstruction [31,37,68,69], as well as for post-traumatic and post-mucormycosis defects [22,24,27]. Several case reports described rehabilitation in syndromic or congenital conditions, including ectodermal dysplasia, cleft lip and palate sequelae, EEC syndrome, and Papillon–Lefèvre syndrome [28,29,33,36]. Other reported indications included patients with a history of medication-related osteonecrosis of the jaw [32], combination with orthognathic surgery such as Le Fort I osteotomy or advancement [29,52], sectional or unilateral rehabilitation [53], and mandibular overdenture retention [23]. Two additional reports described immediate obturation or fixed rehabilitation protocols using a maxillary framework in complex prosthodontic scenarios [26,44].

### 3.5. Clinical Outcomes and Implant Survival

#### 3.5.1. Primary Clinical Evidence

Individual retrospective and multicenter studies reported broadly consistent short- and medium-term outcomes, generally describing high implant and prosthesis survival with a low rate of framework removal, although follow-up periods varied considerably and rarely extended beyond three to five years [42,43,44,45,46,47].

Beyond implant survival, one pilot cohort study specifically evaluated patient-reported quality of life following rehabilitation with customized subperiosteal implants, reporting favorable patient-centered outcomes and supporting the value of incorporating patient-reported outcome measures into future studies of this technique [49].

#### 3.5.2. Secondary Evidence

Secondary evidence was considered separately from the primary clinical literature because the systematic reviews and meta-analyses summarized below include primary studies that may also be represented individually in the present review. Their pooled estimates are therefore presented as higher-level syntheses of the available evidence and were not combined with outcomes from the corresponding primary studies as independent observations.

The systematic review and meta-analysis by Sánchez-Labrador et al. [55], focused specifically on full-arch rehabilitation of severely resorbed edentulous jaws, similarly concluded that subperiosteal implants provide a clinically viable option with favorable short- to medium-term performance, while highlighting the paucity of comparative, long-term data. A dedicated systematic review comparing graftless rehabilitation strategies concluded that both zygomatic and subperiosteal implants can achieve satisfactory outcomes in the severely atrophic maxilla, with the choice between techniques depending on anatomical, prosthetic, and patient-related factors [1].

Among the systematic reviews and meta-analyses included in this synthesis, implant survival in the short-to-medium term was consistently favorable. Anitua et al. [13], pooling data from 13 studies and 227 additively manufactured subperiosteal implants with a weighted mean follow-up of 21.4 months, reported that 97.8% of implants remained in function, with only five reported failures. Al-Nawas and Bär [10], in a systematic review of 24 studies encompassing 246 implants with a mean follow-up of 17 months, similarly reported that 97.6% of implants were functional, with individual study survival rates ranging from 94.1% to 100%. Herce-López et al. [3], in a consensus report and literature review, described biological complication rates ranging from 5.7% to 43.8% and mechanical complication rates ranging from 6.3% to 20% across the reviewed literature, underscoring the wide variability between studies and design protocols.

Longer-term data remain considerably more limited. A recent systematic review and meta-analysis of customized CAD/CAM 3D-printed titanium subperiosteal implants, based on 11 studies comprising 268 patients and 369 implants [14], reported a pooled short-term survival rate of 97.8% for studies with follow-up of three years or less, but an overall pooled survival rate of 92.4% once longer-follow-up studies were incorporated, with the single longest-follow-up study (six years) showing a marked decline in survival to 54.1% (this finding represents the experience of a single cohort and should not be extrapolated to contemporary digitally designed subperiosteal implants as a whole, particularly given the heterogeneity of implant designs, clinical indications, and follow-up periods across the available literature). This finding was interpreted by the review authors as evidence that late, soft-tissue-related complications remain the principal threat to the durability of subperiosteal rehabilitation, and it represents one of the most clinically important signals identified across the reviewed literature.

### 3.6. Complications

Soft-tissue-related complications—most frequently partial exposure or dehiscence of the framework—were the most reported adverse events across the included literature, consistent with findings from both the historical and the digitally manufactured subperiosteal implant experience [10,13,14]. Infectious complications, while less frequent, were reported in a subset of studies and represented, together with soft-tissue exposure, the principal cause of implant removal when failure occurred [3,11]. Given their clinical relevance and their potential influence on long-term implant survival, the presentation, timing, prevention, and management of soft-tissue complications are discussed separately below.

Mechanical and prosthetic complications, including fracture of provisional or definitive restorations and, less frequently, framework-related mechanical issues, were reported across several studies, with rates that varied considerably depending on the prosthetic protocol and loading strategy employed [3,13,41]. The scoping review by Ruiz-Rincón et al. [12], focused specifically on CAD/CAM subperiosteal implants supporting fixed partial restorations, similarly emphasized that prosthetic-related complications, alongside soft-tissue exposure, were among the most frequently reported adverse events in this specific clinical scenario.

#### Management of Soft-Tissue Complications

Customised subperiosteal implants can cause a variety of soft-tissue complications, from limited wound dehiscence and localised mucosal inflammation to progressive framework exposure, suppuration, recurrent infection, and, in more extreme situations, loss of implant stability necessitating framework removal [3,11,13,14]. The most often documented biological consequences in recent series and systematic reviews are soft-tissue dehiscence and subsequent framework exposure [3,13,14,70]. Although there is significant variation across individual studies and clinical protocols, a recent systematic review and meta-analysis that particularly addressed this outcome revealed an overall rate of soft-tissue dehiscence of 28.6% [70].

Although the subperiosteal implant literature currently lacks standardized time-based criteria, soft-tissue complications can be broadly classified as early or late events from a temporal perspective [11,14]. Early complications may include wound dehiscence, localized inflammation, discomfort, edema, or early infection and typically occur during the postoperative healing phase [10,11]. Late complications may include progressive mucosal recession or dehiscence, framework exposure, chronic inflammation, recurrent infection, suppuration, and progressive peri-framework tissue breakdown, and may develop after initial healing and functional loading [3,11,13,14]. Crucially, several clinical series have documented exposed frameworks that continued to function throughout follow-up, indicating that limited asymptomatic exposure does not always indicate rapid implant failure [3,11].

Therefore, the degree and course of the exposure, the existence of inflammation or infection, the state of the surrounding soft tissues, and the stability of the implant should all be taken into consideration when managing the situation [3]. Initially, conservative management of small, stable, asymptomatic exposures without suppuration or increasing tissue disintegration may involve close clinical surveillance, professional cleaning of the exposed framework, removal of accessible plaque-retentive elements, and strengthening of dental hygiene [3,11,71]. If the prosthesis is screw-retained, removing it could make it easier for the exposed surfaces to be thoroughly cleaned by a specialist. Additionally, patients should be told to report any increased redness, bleeding, swelling, pain, or additional exposure as soon as possible [3].

Surgical intervention may be necessary for persistent or increasing dehiscence, recurring inflammation, mucosal ulceration, suppuration, or an exposure that cannot be sufficiently maintained with conservative therapy [3,71]. Treatment options include debridement and local soft-tissue revision, soft-tissue augmentation to increase covering and the amount of keratinised mucosa, or advancement or rearrangement of surrounding tissue, depending on the clinical condition. Although there is currently little evidence to support their routine use, buccal fat pad flaps have also been suggested as a possible treatment for specific maxillary CSI exposures [3,71]. Lastly, when exposure is linked to loss of implant stability, uncontrolled or recurrent infection, progressive tissue disintegration, or failure of conservative and reconstructive procedures, removal of some or all the frameworks should be taken into consideration [3,71].

Since there is still little data to support particular treatment protocols, prevention is still crucial. The risk of soft-tissue breakdown has been suggested to be decreased by appropriate incision design, tension-free closure, preservation or augmentation of keratinised mucosa when necessary, avoidance of sharp framework contours and abrupt transitions around prosthetic connections, precise framework adaptation, and a structured maintenance program [3,71]. However, rather than comparative prospective trials, most recommendations for managing CSI exposure are currently based on retrospective series, case reports, and expert consensus. Standardised criteria that differentiate manageable exposure from clinically significant implant failure have yet to be established.

The proposed management table (Table 2) summarizes currently reported approaches and expert recommendations; it should not be interpreted as a validated treatment algorithm because comparative evidence remains limited.

### 3.7. Comparative and Adjunctive Reconstructive Approaches

Several included studies described the use of customized subperiosteal implants in combination with other reconstructive procedures, including free-flap reconstruction following oncologic ablation [31,68,69], orthognathic surgery [29,52], and, in one report, combined zygomatic and subperiosteal implant strategies for complex post-traumatic and pathologic defects [57]. These combined approaches illustrate the expanding role of digitally designed subperiosteal frameworks as part of a broader reconstructive armamentarium rather than as a stand-alone technique limited to the classically atrophic edentulous patient.

### 3.8. Emerging Directions

A small number of recent publications reflect emerging areas of interest within this field. Gellrich et al. [50] described innovative one-piece, rigidly fixated landing-zone designs intended to further improve primary stability and long-term framework integration in complex maxillary and midfacial defects, while a related perspective article from the same research group discussed personalized rehabilitation strategies for exceptionally complex anatomical scenarios [59]. Meto et al. [65] reported on a fully digital workflow for juxta-osseous implant design specific to severe maxillary atrophy.

## 4. Discussion

The evidence synthesized in this review indicates that the integration of CBCT-based virtual planning, CAD/CAM design, and additive manufacturing has substantially transformed contemporary subperiosteal implantology. However, interpretation of this literature requires consideration of the different methodological roles and levels of evidence represented. Primary clinical evidence remains dominated by case reports, small case series, and retrospective studies, whereas systematic reviews and meta-analyses provide secondary syntheses that partially overlap with these primary publications. Technical and preclinical investigations, in turn, inform biomechanical, material, and manufacturing considerations but do not provide direct evidence of clinical effectiveness [2,3]. Within the secondary evidence, reported pooled short-to-medium-term implant survival has generally ranged from approximately 92% to 98% [10,13,14], which compares favorably with historical reports of cast subperiosteal implants and is broadly comparable to reported outcomes for zygomatic implants in similarly complex clinical scenarios [1].

At the same time, the evidence synthesized in this review highlights several important limitations that should temper enthusiasm for the technique. First, the literature remains dominated by case reports and small case series, which, while valuable for describing novel indications and technical refinements, are inherently limited in their ability to inform robust conclusions about survival and complication rates [19,21,24]. Second, follow-up periods across nearly all included studies remain short, rarely extending beyond three to five years; the marked decline in survival reported at six years in the meta-analysis by Cosola et al. [14] is a particularly important signal that late, soft-tissue-related complications may compromise the long-term durability of even well-planned digital frameworks, and it underscores the urgent need for studies with extended follow-up. Third, considerable heterogeneity exists across the included literature in implant design, manufacturing technology, material selection, prosthetic loading protocol, and outcome definitions, which precluded formal quantitative meta-analysis in this review and has similarly limited the systematic reviews included in this synthesis [3,13].

Soft-tissue exposure and dehiscence emerged consistently as the principal complication associated with digitally designed subperiosteal implants, echoing concerns first raised with historical cast frameworks [10,14]. This recurring finding suggests that, despite substantial improvements in framework fit and design precision afforded by digital planning, the fundamental biological challenge of achieving stable, long-term soft-tissue coverage over a supraperiosteal metallic framework has not yet been fully resolved, and it represents a priority area for future biomaterials and design research, including the lattice and biofunctionalized framework architectures explored in some of the included technical studies [62,63].

The contemporary resurgence of subperiosteal implants should not be interpreted simply as the reintroduction of a historical implant concept using modern manufacturing tools. Rather, digital technologies have modified several of the technical limitations that contributed to the decline of conventional subperiosteal implants. CBCT-based anatomical reconstruction has eliminated the need for direct bone impressions, CAD-based design allows the framework and fixation strategy to be planned before surgery, and additive manufacturing enables the fabrication of complex patient-specific geometries that were difficult to reproduce using conventional casting techniques. Collectively, these developments appear to have improved the reproducibility of the manufacturing workflow and the ability to achieve anatomically customized framework designs [3,4,8,13,39,47,67].

However, technological refinement has not eliminated the principal biological vulnerability of the subperiosteal concept. Soft-tissue dehiscence and framework exposure remain among the most frequently reported complications of contemporary customized subperiosteal implants, and infection may develop when exposure progresses or cannot be adequately maintained [3,11,13,14,16,70,71]. Moreover, although virtual planning and finite-element analysis permit increasingly sophisticated framework optimization, standardized parameters for framework thickness, fixation geometry, surface characteristics, soft-tissue requirements, and prosthetic loading have not yet been established. Thus, improvements in manufacturing precision should not be equated with resolution of the biological and biomechanical challenges that ultimately determine long-term clinical performance [3,11,13,14,16,70,71].

The principal question for the next phase of research is therefore no longer whether customized subperiosteal implants can be digitally designed and manufactured, but in which patients and under which clinical conditions they provide a predictable advantage over established alternatives. Current evidence remains insufficient to define validated patient-selection criteria, standardized indications relative to augmentation or zygomatic implant therapy, optimal framework and surface characteristics, minimum soft-tissue requirements, or evidence-based algorithms for the management of framework exposure [2,3,8,13,14]. Furthermore, robust long-term survival data, patient-reported outcomes, and comparative prospective studies remain scarce. These gaps currently represent a greater limitation to widespread clinical adoption than the technical feasibility of manufacturing the implant itself [2,10,15,16,55].

The apparent improvements summarized in Table 3 reflect the direction of the available evidence and should not be interpreted as proof of superiority, as direct comparative evidence between historical and contemporary workflows remains limited.

This critical perspective also differentiates the present review from recently published syntheses. Albash et al. provided a broad state-of-the-art narrative overview of customized subperiosteal dental implants, whereas Vaira et al. mapped the expanding spectrum of patient-specific subperiosteal implants across established and emerging clinical indications [15,16]. The present review deliberately narrows its focus to severe jaw atrophy and uses this clinical setting to examine the translation of technological progress into clinical performance, with particular attention to persistent complications, their management, therapeutic positioning, and unresolved evidence gaps. Thus, its purpose is not to provide a broader catalogue of applications, but to critically assess how far the “digital renaissance” has translated into resolution of the limitations historically associated with subperiosteal implant therapy.

The expanding range of clinical indications identified in this review—extending well beyond the classic atrophic-edentulism scenario to include oncologic, post-traumatic, syndromic, and orthognathic-combined cases [22,28,29,31,36,68,69]—illustrates the versatility of the digital subperiosteal implant workflow, but also further complicates efforts to draw generalizable conclusions about outcomes, since these diverse patient populations differ substantially in bone quality, soft-tissue condition, and functional demands. Future studies would benefit from stratifying outcomes according to these distinct clinical scenarios rather than reporting pooled results across a heterogeneous patient population.

In addition, some secondary evidence sources included primary studies that were also represented individually in the reviewed literature, and some clinical publications may have originated from overlapping patient cohorts. Publication counts should therefore not be interpreted as numbers of independent clinical datasets or patient populations. To minimize the risk of double counting, primary clinical evidence, secondary evidence, and technical/preclinical evidence were interpreted separately, and pooled estimates from secondary studies were not combined with outcomes from their constituent primary studies.

Finally, patient-centered outcomes remain markedly underrepresented in the current literature, with only one identified pilot study formally assessing quality of life after subperiosteal implant rehabilitation [49]. Given the complexity and cost of these interventions, incorporating validated patient-reported outcome measures into future prospective studies should be considered a priority.

The search was restricted to PubMed and Scopus, supplemented by manual searching, and was limited to English-language publications. Although these databases were selected for their complementary biomedical, dental, and multidisciplinary coverage, the absence of additional bibliographic databases such as Embase may have resulted in relevant studies being missed, particularly case reports or case series published in journals with more limited indexing. This potential selection bias should be considered when interpreting the present synthesis. A formal risk-of-bias or methodological quality assessment was not performed, consistent with the narrative, thematic nature of this synthesis rather than a formal systematic review with meta-analysis. Finally, because the review draws on studies with markedly different designs, follow-up durations, and outcome definitions, the survival and complication figures reported here should be interpreted as indicative of overall trends rather than as precise, directly comparable estimates.

## 5. Conclusions

Digitally designed, patient-specific subperiosteal implants represent a renewed treatment approach for selected patients with severe maxillary or mandibular atrophy in whom conventional endosseous implant rehabilitation may require extensive reconstructive procedures or may not be feasible. Advances in three-dimensional imaging, virtual planning, CAD/CAM design, and additive manufacturing have enabled more individualized implant geometries and digitally supported surgical workflows. Available clinical reports suggest encouraging short- to medium-term outcomes; however, the current evidence remains predominantly observational, heterogeneous, and based largely on case reports, small case series, and retrospective studies. Long-term outcome data remain limited, and the available evidence suggests that survival may decline substantially beyond five years, with the longest-follow-up study included in this review reporting an estimated six-year survival of 54.1%. However, this finding derives from a single clinical cohort and should not be generalized across different implant designs, indications, and clinical protocols. Consequently, digitally customized subperiosteal implants should presently be regarded as a promising option for carefully selected cases rather than as an established equivalent to conventional implant rehabilitation. Prospective multicenter studies using standardized definitions of survival, success, complications, and patient-reported outcomes, together with longer follow-up, are required to establish their long-term predictability and clinical effectiveness.

## Figures and Tables

**Table 1:** Distribution of the core literature informing the narrative synthesis by publication/study design.

Evidence Domain	Study Category	Number of Studies (*n* = 62)
Primary clinical evidence	Clinical studies, retrospective/cohort/multicenter/pilot studies, case series, case reports, and clinical reports	38
Secondary and contextual evidence	Systematic review/meta-analysis/scoping review	10
	Narrative review/consensus/perspective article, and mixed review–clinical reports	9
Technical/preclinical evidence	Finite-element, biomechanical, materials, and methodology-focused studies	5

**Table 2:** Clinical presentation and management of soft-tissue complications associated with customized subperiosteal implants.

Timing/Presentation	Typical Findings	Initial Management	Escalation
Early wound dehiscence	Incision opening, inflammation, swelling	Hygiene optimization, professional cleaning, close monitoring	Debridement/soft-tissue revision if progressive
Limited stable exposure	Small exposed framework, no suppuration or mobility	Professional cleaning, hygiene reinforcement, monitoring	Surgery if exposure progresses
Persistent inflammatory exposure	Redness, bleeding, ulceration, plaque retention	Debridement, prosthetic access where possible	Soft-tissue revision/augmentation
Infection	Suppuration, pain, recurrent swelling	Local debridement ± appropriate infection management	Surgical revision/removal if uncontrolled
Advanced/late failure	Progressive exposure, recurrent infection, implant mobility	—	Partial/complete framework removal

**Table 3:** Critical discussion on historical limitations, contemporary digital solutions, and unresolved challenges of customized subperiosteal implants.

Domain	Historical Limitation	Contemporary Digital Solution	Apparent Improvement	Remaining Limitation/Evidence Gap
Anatomical acquisition	Direct surgical bone impression	CBCT-based 3D reconstruction	Non-invasive preoperative anatomical acquisition	Segmentation and imaging accuracy remain relevant
Framework fabrication	Lost-wax casting and manufacturing inaccuracies	CAD + DMLS/SLM	Greater design reproducibility and geometric freedom	Process-dependent dimensional variation and post-processing
Framework adaptation	Difficult passive adaptation	Patient-specific CAD design	Improved planned anatomical conformity	No standardized clinical threshold for acceptable fit
Biomechanics	Empirical framework design	Virtual planning and FEA	Preoperative optimization of geometry/stress distribution	No validated universal design parameters
Soft tissues	Dehiscence and framework exposure	Improved contouring, soft-tissue planning and maintenance	Better prevention/management possibilities	Exposure remains a major complication
Infection	Difficult management after exposure	Earlier surveillance and structured maintenance	Potential for earlier intervention	Recurrent infection may still require removal
Clinical outcomes	Declining historical long-term survival	Contemporary customized CSI	Encouraging short-/medium-term outcomes	Limited robust long-term evidence
Patient selection	Primarily salvage indication	Digital multidisciplinary planning	More individualized treatment planning	No validated indication/selection algorithm

## Data Availability

The original contributions presented in this study are included in the article or Supplementary Materials. Further inquiries can be directed to the corresponding authors.

## Supplementary Materials

The following supporting information can be downloaded at: https://www.mdpi.com/article/10.3390/dj14100685/s1. Table S1: Search strategy and study-level record of the publications assessed during the literature-selection process, including the predefined criteria applied to determine their inclusion in the narrative synthesis.

## Abbreviations

The following abbreviations are used in this manuscript:

AI	Artificial Intelligence
CAD/CAM	Computer-Aided Design/Computer-Aided Manufacturing
CBCT	Cone-Beam Computed Tomography
CSI	Customized Subperiosteal Implant
DMLS	Direct Metal Laser Sintering
EEC	Ectrodactyly–Ectodermal Dysplasia–Cleft Syndrome
PEEK	Polyetheretherketone
PEKK	Polyetherketoneketone
PRISMA	Preferred Reporting Items for Systematic Reviews and Meta-Analyses
SLM	Selective Laser Melting
